# Sarcopenia in Colorectal Cancer Surgery—Minimally Invasive vs. Open

**DOI:** 10.1002/jcsm.70065

**Published:** 2025-09-16

**Authors:** Felix Merboth, Miriam Müller‐Oerlinghausen, Heiner Nebelung, Andreas Bogner, Mathieu Pecqueux, Nadja Salisch, Marius Distler, Verena Plodeck, Ralf‐Thorsten Hoffmann, Johannes Fritzmann, Jürgen Weitz, Johanna Kirchberg

**Affiliations:** ^1^ Department of Visceral, Thoracic and Vascular Surgery University Hospital and Faculty of Medicine Carl Gustav Carus, Technische Universität Dresden Dresden Germany; ^2^ National Center for Tumor Diseases (NCT/UCC) Dresden Germany; ^3^ German Cancer Research Center (DKFZ) Heidelberg Germany; ^4^ University Hospital and Faculty of Medicine Carl Gustav Carus, Technische Universität Dresden Dresden Germany; ^5^ Institute and Polyclinic for Diagnostic and Interventional Radiology University Hospital Carl Gustav Carus, Technical University Dresden Dresden Germany; ^6^ General, Visceral and Transplant Surgery, Department of Surgery Medical University of Graz Graz Austria

**Keywords:** minimally invasive surgery, oncological outcome, open surgery, rectal cancer, sarcopenia, skeletal muscle index

## Abstract

**Background:**

Sarcopenia, characterized by loss of skeletal muscle mass and strength, is prevalent in patients undergoing treatment for colorectal cancer. Sarcopenia's prevalence in patients with cancer can reach up to 50% and is known to exacerbate postsurgical complications and affect long‐term oncological outcomes. This study examined whether minimally invasive surgery (MIS) offers protective benefits against postoperative sarcopenia compared with open surgery in patients undergoing rectal cancer resection.

**Methods:**

This retrospective analysis included 145 patients who underwent open or minimally invasive (laparoscopic or robot‐assisted) rectal resections at the University Hospital Dresden between 2013 and 2021. Confounding variables were adjusted using propensity score matching. The skeletal muscle index (SMI) and psoas muscle thickness per height (PMTH) were analysed in preoperative and postoperative computed tomography scans to measure changes in skeletal muscle mass. Potential risk factors for muscle loss were evaluated, and oncological long‐term outcome was analysed.

**Results:**

The results indicate that oncological rectal resection did not result in pronounced postoperative muscle loss. No significant difference between the open and MIS groups in terms of postoperative muscle loss over 3 years postoperatively could be detected. Wound healing disorders were identified as the most significant independent risk factors for muscle loss (SMI loss > 10%). In contrast, neither the type of surgical technique nor the presence of a protective loop ileostomy significantly influenced the development of postoperative muscle loss.

Patients who experienced a > 10% SMI loss within the first year had significantly poorer overall and disease‐free survival. The 1‐year survival rate was 93.3% in the group with high SMI loss compared with 100.0% in the group with low SMI loss (*p* = 0.435). The 3‐year (66.7% vs. 95.6%, HR 8.75, 95% CI 1.855–41.286, *p* = 0.006) and 5‐year (44.4% vs. 93.3%, HR 11.072, 95% CI 2.414–50.782, *p* = 0.002) survival rates were significantly lower in patients with high SMI loss. Patients with high SMI loss had an increased likelihood of recurrence and metastasis.

**Conclusions:**

Although MIS did not confer a protective advantage against postoperative muscle loss in patients with rectal cancer, the findings highlight the critical role of maintaining muscle mass in improving survival outcomes. Postoperative muscle loss appears to be a marker of aggressive tumour behaviour, and interventions aimed at minimizing muscle loss, such as enhanced nutritional support, may improve the long‐term patient prognosis. Future studies should explore interventional strategies to mitigate sarcopenia in this population.

## 1 Introduction

Colorectal carcinoma is among the most common tumours worldwide and accounted for 10% of global cancer incidence and 9.4% of cancer‐related deaths in 2020 [1]. It is the second leading cause of cancer‐related deaths worldwide (WHO, 2023, Colorectal cancer).

Sarcopenia is defined as low skeletal muscle mass and strength by international working groups, such as the Asian Working Group for Sarcopenia and the European Working Group on Sarcopenia in Older People [2]. Sarcopenia has a prevalence of approximately 40%–50% in newly diagnosed patients with cancer [3, 4]. Systemic and surgical therapies further promote catabolic metabolic changes, leading to muscle mass loss [5].

Muscle mass can be assessed and quantified on computed tomography (CT) scans as skeletal muscle index (SMI, cm/m^2^) [6] or psoas muscle thickness per height (PMTH, mm/m) [7]. The roles of SMI and PMTH in measuring muscle mass and their high reproducibility and predictive potential for adverse perioperative and oncological outcomes have been described for many tumour types [8, 9] including rectal cancer [10, 11].

In 2023, we demonstrated for the first time that, in the upper gastrointestinal tract, the robotic surgical approach for oesophageal resection was the only significant protective factor against the development of postoperative muscle mass loss compared with open oesophageal resection [12]. There is further evidence that the loss of muscle mass within 7 days postoperatively is a parameter for worsened long‐term survival [13].

These results are of high clinical relevance because the development of sarcopenia can be mitigated. Perioperative and oncological outcomes potentially improve after enforcing enhanced recovery after surgery and prehabilitation programmes, targeted nutritional therapies and the use of a minimally invasive surgical procedure [11, 12, 14].

However, whether minimally invasive (laparoscopic or robot‐assisted) rectal resection also protects against the development of postoperative muscle mass loss compared with open surgery has not yet been investigated.

This study compared postoperative changes in skeletal muscle mass (SMI and PMTH) within 36 months after rectal resection between open and minimally invasive surgical techniques. Univariate and multivariate analyses evaluated the potential factors influencing the loss of skeletal muscle mass and oncological outcomes.

We hypothesized that minimally invasive rectal resection is associated with less postoperative muscle mass loss than open rectal resection and may have a positive influence on oncological outcomes and perioperative complications.

## 2 Methods

All patients who underwent open or minimally invasive (laparoscopic or robot‐assisted) rectal resection at the Department of Visceral, Thoracic, and Vascular Surgery at University Hospital Dresden between 2013 and 2021 were included in this retrospective analysis. Inclusion criteria were (1) biopsy‐confirmed rectal or rectosigmoid carcinoma, (2) planned oncological rectal resection with anastomosis with/without loop ileostomy and (3) availability of at least one preoperative and postoperative CT scan.

Exclusion criteria were benign histopathology, emergency operations, rectal resection without primary anastomosis, other active malignant tumours within 5 years before rectal carcinoma, lack or insufficient quality of preoperative or postoperative CT scans and patients' death in hospital or within the first 30 days postoperatively.

The study protocol was reviewed by the local ethics committee (BO‐EK‐98022023) and was performed in accordance with the Declaration of Helsinki and its later amendments.

### 2.1 Oncological Staging and Treatment Algorithms

According to the guidelines of the local Comprehensive Cancer Center (National Center for Tumor Diseases), staging workups for all patients with histologically proven rectal cancer included rectoscopy; CT scan of the thorax, abdomen and pelvis; and rectal magnetic resonance imaging (MRI). After completing these staging examinations, all patients were reviewed at a multidisciplinary tumour conference (MTB).

Neoadjuvant treatment was indicated when clinical staging revealed infiltration of or close contact with the mesorectal fascia (< 2 mm), suspicious lateral lymph nodes, extramural vascular invasion or involvement of or close contact with the musculus sphincter ani externus.

The standard neoadjuvant chemoradiation regimen consisted of 50.4 Gy, capecitabine/5‐fluorouracil and rectal resection after 4–6 weeks or 5 × 5 Gy and rectal resection after 7 days or 6 weeks. In October 2020, the standard regimen was changed to total neoadjuvant therapy according to the RAPIDO trial [15].

Standard restaging CT and rectal MRI were performed after completion of neoadjuvant therapy. Subsequently, the MTB recommended surgery when restaging CT scans did not reveal any new distant metastases or other contraindications. After surgery, the final histology was again discussed by the MTB.

Adjuvant chemotherapy was not regularly recommended for patients who completed neoadjuvant chemoradiation. In cases of resection without neoadjuvant therapy, adjuvant treatment was discussed individually for extensive disease. All patients underwent standardized routine follow‐up examinations (rectoscopy and CT scan of the thorax, abdomen and pelvis) every 6 months for 5 years.

### 2.2 Surgical Technique

Open, minimally invasive and robotic resections were performed by a trained team of surgeons according to standard algorithms as described previously [16].

In cases of clinically relevant preoperative ileus, colostomy or ileostomy was established before the initiation of neoadjuvant therapy. A protective loop ileostomy was established intraoperatively in cases of total mesorectal excision and anastomosis of the lower rectum. Feeding tubes were not routinely used for patients with rectal cancer.

### 2.3 Assessment of Skeletal Muscle Mass

In this study, we solely analysed the skeletal muscle mass component of sarcopenia and not the skeletal muscle strength component. Skeletal muscle mass was assessed by two independent, experienced investigators who were blinded to the time point of the CT scan and the surgical approach. If no CT scans were available at the exact time point, the CT scan closest to the defined time point was selected for analysis. The skeletal muscle area (SMA; cm^2^) was measured on an axial CT scan at the level of the L3 vertebral body using the IMPAX tool (IMPAX EE R20; Agfa HealthCare, Mortsel, Belgium) to draw a contour with automatically supported edge detection.

SMA was normalized to the square of the patient's height (m^2^) to calculate SMI (cm/m^2^). The transverse psoas muscle thickness (mm) was evaluated on an axial CT scan at the level of the umbilicus. It was defined as the transverse diameter of the psoas muscle perpendicular to the largest diameter of the muscle in the axial view. Transverse psoas muscle thickness was normalized to the patient's height (m) to calculate PMTH (mm/m) [7, 17].

Sarcopenia was defined as SMI < 38.5 cm/m^2^ for women and < 52.4 cm/m^2^ for men according to Prado et al. [18], and as PMTH < 10.4 mm/m for women and < 17.3 mm/m for men according to Gu et al. [7]. A relevant loss of muscle mass was defined as an SMI loss of > 10% within the first postoperative year compared with the preoperative value [19].

### 2.4 Outcome Measures

The primary outcome was longitudinal postoperative changes in skeletal muscle mass (SMI and PMTH) within 36 months postoperatively by comparing open and minimally invasive surgical techniques.

Secondary outcomes were risk factors for the development of SMI loss within 12 months postoperatively, and the influence of surgical technique and SMI loss on long‐term oncological outcomes.

### 2.5 Statistical Analysis

Statistical analyses were performed using the Statistical Package for the Social Sciences software (SPSS, version 28.0; IBM Corp., Armonk, NY, USA) and Python (version 3.12.1; Python Software Foundation, Beaverton, USA). Continuous variables are presented as mean (± standard deviation) or median with an interquartile range. Continuous data were compared using Student's *t*‐test when the variables were normally distributed. Continuous nonparametric variables were compared using the Mann–Whitney *U* test. One‐way analysis of variance was used to compare longitudinal variations in continuous variables. Categorical variables were compared using the chi‐square or Fisher's exact test. The significance level was set at a *p*‐value equal to 0.05. Univariate and multivariate analyses for loss of muscle mass [7, 18] were performed using binary logistic regression. Variables with *p*‐values of < 0.05 in univariate analysis were included in the multivariate analysis. Overall survival (OS) and disease‐free survival (DFS) were analysed with Cox regression and Log‐rank test and were represented as Kaplan–Meier curves.

To ensure better comparability between the groups, 1‐to‐1 propensity score matching was performed. The following variables were used to calculate the propensity score using the regression models: sex, age, body mass index (BMI), American Society of Anaesthesiologists Classification (ASA), preoperative SMI, presence of a protective loop ileostomy, postoperative severity of complications using the Clavien–Dindo Classification, neoadjuvant treatment, adjuvant treatment and pathologic Union Internationale Contre le Cancer (UICC) stage. Subsequently, the nearest neighbour method with a calibre width of 0.1 was used to find matching pairs.

### 2.6 Terminology

By definition, the term ‘sarcopenia’ is used when referring to the combination of loss of skeletal muscle mass and strength. Otherwise, the terms ‘(skeletal) muscle mass loss’ or ‘skeletal muscle strength loss’ are used explicitly.

## 3 Results

### 3.1 Patient Characteristics

A total of 145 patients met the inclusion criteria during the study period (2013–2021). Of these, 74 (51.0%) underwent open rectal resection and 71 (49.0%) underwent minimally invasive rectal resection (MIS). In the MIS group, 48 (67.6%) of the operations were robot‐assisted. The average age of the cohort was 61.6 years, and 44 (30.3%) patients were female.

Patient baseline characteristics, including age, BMI, histology and perioperative complications, were comparable between the open surgery and MIS groups (Table S1). However, there were significantly more ASA 3 patients in the open cohort than in the MIS group (33 [44.6%] vs. 20 [28.2%], *p* = 0.008). Neoadjuvant therapy consisted of radiochemotherapy in 56 (38.6%), chemotherapy in 9 (6.2%) and radiotherapy in 5 (3.4%) patients. In the MIS group, significantly more patients did not receive neoadjuvant therapy (47 [66.2%] vs. 28 [37.8%], *p* = 0.001) and fewer patients were in UICC Stage IV (6 [8.5%] vs. 24 [32.4%], *p* = 0.002).

A loop ileostomy was performed intraoperatively in 58 (78.4%) and 56 (78.9%) patients in the open and MIS group (*p* = 1.000), respectively. It was taken down within 6 months in 19 (25.7%) and 24 (33.8%) patients, respectively (*p* = 0.42). In both groups, the rates of anastomotic leak (16 [21.6%] vs. 12 [16.9%], *p* = 0.532) and wound infection (11 [14.9%] vs. 5 [7.0%], *p* = 0.186) did not differ significantly.

In both the groups, the muscle mass indices, SMI (43.8 ± 8.6 cm/m^2^ vs. 45.0 ± 8.3 cm/m^2^, *p* = 0.435) and PMTH (19.1 ± 4.1 mm/m vs. 19.7 ± 3.3 mm/m, *p* = 0.246), were not significantly different preoperatively. According to Prado's classification [6], 75.7% of the patients in the open group were classified as sarcopenic preoperatively compared with 71.7% in the MIS group (*p* = 0.708).

After matching, the open surgery and MIS groups each comprised 45 patients. In the MIS group, laparoscopic and robotic surgeries were performed in 15 (33.3%) and 30 (66.7%) patients, respectively. In contrast to the unmatched cohort, the patient characteristics as well as the surgical and histopathological findings were comparable (Table 1).

**TABLE 1:** Patient characteristics, histopathologic and surgical findings.

	Open	MIS	*p*
	*n* = 45	*n* = 45	
Age [years]	60.3 (±11.3)	62.9 (±9.2)	0.280^a^
Sex			0.638
Female	11 (75.6)	14 (31.1)	
Male	34 (75.6)	31 (68.9)	
BMI [kg/m^2^]	26.9 (±4.5)	26.5 (±3.7)	0.998^a^
ASA			
1	1 (2.2)	1 (1.1)	0.828
2	26 (57.8)	29 (64.4)	
3	18 (40.0)	15 (33.3)	
Neoadjuvant treatment			0.474
None	21 (46.7)	27 (60.0)	
Chemotherapy	1 (2.2)	1 (2.2)	
Chemoradiotherapy	23 (51.1)	17 (37.8)	
Adjuvant treatment			0.671
None	24 (53.3)	26 (57.8)	
Chemotherapy	21 (46.7)	18 (40.0)	
Chemoradiotherapy	0 (0.0)	1 (2.2)	
pT stage			0.764
0	3 (6.7)	2 (4.4)	
1	6 (13.3)	5 (11.1)	
2	16 (35.6)	17 (37.8)	
3	18 (40.0)	21 (46.7)	
4	2 (4.4)	0 (0.0)	
pN stage			0.317
0	28 (62.2)	29 (64.4)	
1	16 (35.6)	12 (26.7)	
2	1 (2.2)	4 (8.9)	
pM stage			0.384
0	36 (80.0)	40 (88.9)	
1	9 (20.0)	5 (11.1)	
UICC stage			0.705
1	17 (37.8)	20 (44.4)	
2	8 (17.8)	9 (20.0)	
3	11 (24.4)	11 (24.4)	
4	9 (20.0)	5 (11.1)	
Loop ileostomy			1.000
No	9 (20.0)	10 (22.2)	
Yes	36 (80.0)	35 (77.8)	
Loop ileostomy closure			0.670
< 6 months	15 (41.7)	18 (51.4)	
> 6 months	13 (36.1)	12 (34.3)	
Never	8 (22.2)	5 (14.3)	
Minor complications	8 (17.8)	5 (11.1)	0.550
Major complications	12 (26.7)	15 (33.3)	0.646
Anastomotic leakage	12 (26.7)	8 (17.8)	0.447
Ureteral lesion	0 (0.0)	1 (2.2)	1.000
Others	0 (0.0)	6 (13.3)	0.026
CDC > 2	12 (26.7)	14 (31.1)	0.816
In hospital mortality	0 (0.0)	0 (0.0)	1.000
30‐day mortality	0 (0.0)	0 (0.0)	1.000
Length of hospital stay [d]	17.7 (±13.1)	16.2 (±11.6)	0.140^a^

*Note:* n (%), mean (± standard deviation) or median (IQR), Fisher's exact test.

Abbreviations: ASA, American Society of Anaesthesiologists Classification; BMI, body mass index; CDC, Clavien–Dindo Classification; MIS, minimally invasive surgery.

^a^ Mann–Whitney *U* test.

### 3.2 Course of Muscle Mass Within the First Three Postoperative Years Depending on the Surgical Technique

A total of 629 CT scans were analysed, corresponding to an average of 4.3 per patient.

**FIGURE 1:**
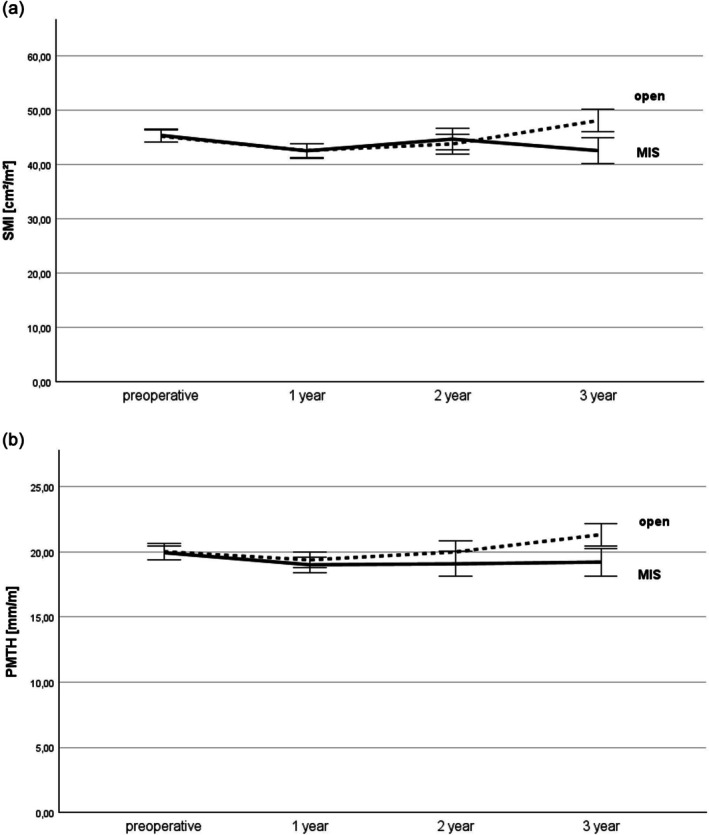
Course of muscle mass measured by (a) skeletal muscle index (SMI) and (b) psoas muscle thickness per height (PMTH) within the first three postoperative years depending on the surgical technique. The muscle mass remains stable over 3 years postoperatively, regardless of the surgical approach. Open group: dotted line, MIS group: continuous line.

In the matched cohort, preoperative SMI or PMTH were not significantly different between the open and MIS groups (45.2 ± 7.6 cm/m^2^ vs. 45.4 ± 8.2 cm/m^2^, *p* = 0.97; 20.0 ± 4.1 mm/m vs. 19.9 ± 3.7 mm/m, *p* = 0.97). In both groups, SMI and PMTH remained almost completely stable over the first 3 years postoperatively (Figure 1 and Table 2). No difference in SMI was observed between the open and MIS groups in postoperative year one (42.6 ± 8.1 cm/m^2^ vs. 42.6 ± 8.8 cm/m^2^, *p* = 0.99), two (43.8 ± 9.4 cm/m^2^ vs. 44.7 ± 8.3 cm/m^2^, *p* = 0.70) or three (48.1 ± 9.5 cm/m^2^ vs. 42.6 ± 9.0 cm/m^2^, *p* = 0.19). Similarly, there were no differences in PMTH between the open and MIS groups in postoperative year one (19.4 ± 3.8 mm/m vs. 19.0 ± 3.7 mm/m, *p* = 0.70), two (20.0 ± 4.5 mm/m vs. 19.1 ± 3.9 mm/m, *p* = 0.56) or three (21.3 ± 3.9 mm/m vs. 19.2 ± 4.1 mm/m, *p* = 0.14).

**TABLE 2:** Evaluation of postoperative muscle mass depending on surgical technique.

	Preoperative			1A postoperative			2A postoperative			3A postoperative		
	Open (*n* = 45)	MIS (*n* = 45)	*p*	Open (*n* = 40)	MIS (*n* = 41)	*p*	Open (*n* = 26)	MIS (*n* = 17)	*p*	Open (*n* = 20)	MIS (*n* = 15)	*p*
SMI [cm^2^/m^2^]	45.2 (±7.6)	45.4 (±8.2)	0.968^a^	42.6 (±8.1)	42.6 (±8.8)	0.994^a^	43.8 (±9.4)	44.7 (±8.3)	0.703^a^	48.1 (±9.5)	42.6 (±9.0)	0.191^a^
Sarcopenic Prado et al.	33 (73.3)	33 (73.3)	1.000	33 (82.5)	33 (80.5)	1.000	21 (80.8)	10 (58.8)	0.168	16 (80.0)	9 (60.0)	0.266
SMI loss [%]	—	—	—	5.7	6.0	0.899^a^	5.3	1.6	0.330^a^	2.4	2.8	0.681^a^
PMTH [mm/m]	20.0 (±4.1)	19.9 (±3.7)	0.966^a^	19.4 (±3.8)	19.0 (±3.7)	0.704^a^	20.0 (±4.5)	19.1 (±3.9)	0.563^a^	21.3 (±3.9)	19.2 (±4.1)	0.139^a^
Sarcopenic Gu et al.	5 (11.1)	5 (11.1)	1.000	7 (17.5)	5 (12.2)	0.547	5 (19.2)	3 (16.7)	1.000	3 (15.0)	1 (6.7)	0.619
PMTH loss [%]	—	—	—	2.8	3.6	0.494^a^	1.4	0.1	0.495^a^	3.3	0.6	0.219^a^

*Note:* n (%), mean (± standard deviation); Fisher's exact test.

Abbreviations: A, year; MIS, minimally invasive surgery; PMTH, psoas muscle thickness per height; SMI, skeletal muscle index.

^a^ Mann–Whitney *U* test.

### 3.3 Risk Factors for Loss of Muscle Mass Within the First Postoperative Year

The univariate analysis of the overall cohort showed that older age (odds ratio [OR] = 1.040, 95% confidence interval [CI]: 1.004–1.076, *p* = 0.029), higher ASA score (OR = 1.961, 95% CI: 1.077–3.570, *p* = 0.028), minor complications (OR = 8.294, 95% CI: 2.504–27.472, *p* < 0.001), major complications (OR = 2.357, 95% CI: 1.098–5.060, *p* = 0.028) and prolonged hospitalization (OR = 1.054, 95% CI: 1.019–1.091, *p* = 0.003) were associated with a relevant loss of muscle mass (defined as SMI loss > 10%) within the first postoperative year. Among the major complications, anastomotic leak was found to be a significant risk factor for the development of SMI loss (OR = 3.822, 95% CI: 1.624–8.997, *p* = 0.002).

In the multivariate analysis of the overall cohort, the presence of minor complications, consisting only of wound healing disorders in this cohort (OR = 4.229, 95% CI: 1.101–16.234, *p* = 0.036), was the only independent factor associated with a relevant loss of muscle mass (Table 3).

**TABLE 3:** Univariate and multivariate analysis for postoperative SMI loss > 10%.

	Univariate			Multivariate		
*n* = 145	OR	95% CI	*p*	OR	95% CI	*p*
Age	**1.040**	**1.004–1.076**	**0.029**	1.020	0.982–1.060	0.306
Sex (f:m)	0.851	0.394–1.839	0.682			
BMI	1.071	0.980–1.170	0.131			
ASA	**1.961**	**1.077–3.570**	**0.028**	1.407	0.722–2.740	0.316
Neoadjuvant treatment	0.855	0.664–1.100	0.222			
Adjuvant treatment	0.606	0.320–1.148	0.124			
UICC stage	1.019	0.748–1.387	0.906			
Ileostomy	1.457	0.596–3.562	0.410			
Ileostomy closure	1.218	0.739–2.006	0.439			
Minor complications	**8.294**	**2.504–27.472**	**< 0.001**	**4.229**	**1.101–16.234**	**0.036**
Major complications	**2.357**	**1.098–5.060**	**0.028**	1.180	0.408–3.410	0.761
CDC > 2	1.774	0.811–3.881	0.151			
Open vs. MIS	0.569	0.279–1.160	0.121			
Length of hospital stay [day]	**1.054**	**1.019–1.091**	**0.003**	1.025	0.977–1.074	0.316
Sarcopenic preoperative (Prado et al.)	1.023	0.461–2.269	0.955			

*Note:* Binary logistic regression; OR, odds ratio; 95% CI, 95% confidence interval.

Abbreviations: ASA, American Society of Anaesthesiologists; BMI, body mass index; CDC, Clavien–Dindo Classification; MIS, minimally invasive surgery.

Wound healing disorders were conservatively treated in 10 out of 16 patients (62.5%) and required surgery in six (37.5%) patients. Five (31.3%) patients underwent vacuum‐assisted closure.

Neoadjuvant (*p* = 0.222) or adjuvant (*p* = 0.124) treatment, as well as UICC stage (*p* = 0.906), intraoperative placement of an ileostomy (*p* = 0.410) and minimally invasive surgical technique (*p* = 0.121), were not associated with postoperative SMI loss.

### 3.4 Influence of Surgical Technique on Oncological Outcomes

The median follow‐up period for survival analyses was 55 (31–66) months.

**FIGURE 2:**
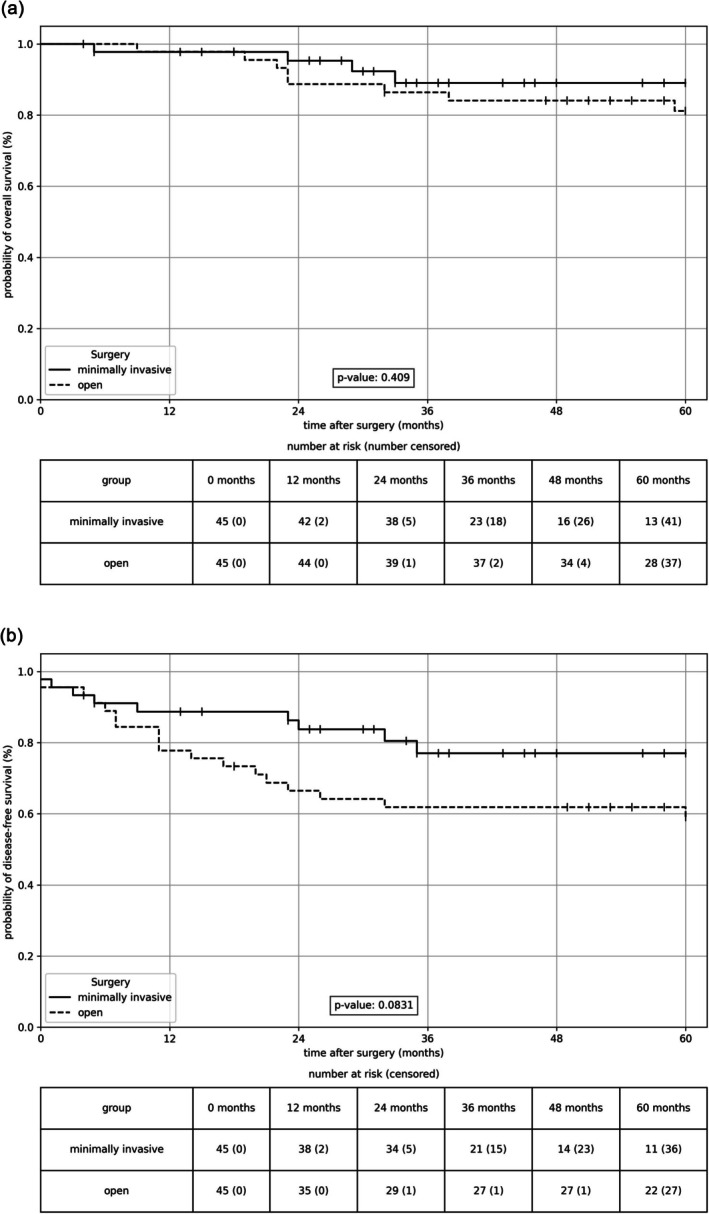
(a) Overall survival and (b) disease‐free survival depending on the surgical approach displayed as Kaplan–Meier curves. There are no significant differences between the two groups in either OS (*p* = 0.409) or DFS (*p* = 0.083). Log rank test.

In the Kaplan–Meier analysis, OS did not differ between the two groups (*p* = 0.409; Figure 2a). The 1‐year survival rates were 97.8% and 97.7% in the open and MIS groups, respectively (HR 1.059, 95% CI 0.066–16.930, *p* = 0.968). In addition, the 3‐year (86.0% vs. 85.2%, HR 1.046, 95% CI 0.295–3.798, *p* = 0.944) and 5‐year (75.8% vs. 75.0%, HR 1.045, 95% CI 0.315–3.472, *p* = 0.943) survival rates did not differ significantly between the two groups.

As shown in the Kaplan–Meier analyses, DFS was slightly but not significantly worse in the open group (*p* = 0.083; Figure 2b). Thus, the surgical technique had no significant effect on the overall or DFS.

### 3.5 Influence of Postoperative Loss of Muscle Mass on Oncological Outcomes

**FIGURE 3:**
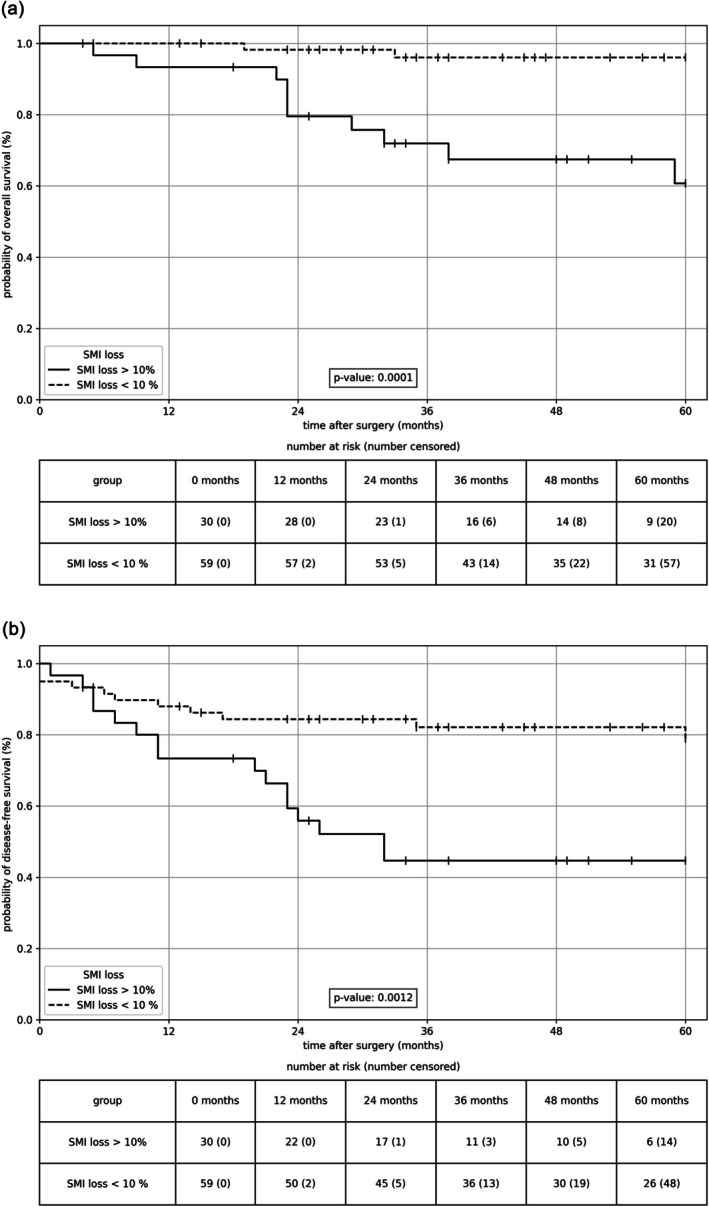
(a) Overall survival and (b) disease‐free survival depending on SMI loss displayed as Kaplan–Meier curves. Patients with a postoperative SMI loss > 10% have a significantly worse OS (*p* < 0.001) and DFS (*p* = 0.001). Log rank test.

Patients with SMI loss > 10% within 12 months postoperatively had a significantly worse OS in the Kaplan–Meier analysis compared with patients with a SMI loss < 10% (*p* < 0.001; Figure 3a). The 1‐year survival rate was 93.3% in the group with high SMI loss compared with 100.0% in the group with low SMI loss (HR n.a., *p* = 0.435), (the 3‐year: 66.7% vs. 95.6%, HR 8.75, 95% CI 1.855–41.286, *p* = 0.006; and the 5‐year: 44.4% vs. 93.3%, HR 11.072, 95% CI 2.414–50.782, *p* = 0.002).

In the Kaplan–Meier analysis, DFS was significantly reduced in patients with high SMI loss (*p* < 0.001; Figure 3b). Recurrence rates differed between the two groups. In the first year after surgery, 23.3% of patients with high SMI loss developed recurrence or metachronous metastases. In the group with low SMI loss, only 11.9% of patients developed recurrence or metachronous metastases (*p* = 0.218). The differences were the same in the second postoperative year (23.1% vs. 15.8%, *p* = 0.157) and became increasingly significant in the third postoperative year (39.1% vs. 17.0%, *p* = 0.045).

Thus, high SMI loss within the first postoperative year after rectal resection was significantly associated with reduced overall and DFS. In addition, the rates of recurrence and metachronous metastasis were considerably higher in patients with high SMI loss than those with low SMI loss.

## 4 Discussion

Sarcopenia is defined as the reduction in skeletal muscle mass and strength. SMI determined using CT has been proven to be the gold standard for determining muscle mass, particularly in patients with cancer. However, standardized cutoff values, as proposed by the European Working Group on Sarcopenia, are still lacking [2]. However, in abdominal cancer surgery, it has been shown that even a postoperative decrease in muscle mass is associated with deterioration in OS [20].

In our study, we showed that, in contrast to oesophageal resection, oncological rectal resection does not result in pronounced postoperative skeletal muscle loss.

Our study is the first to investigate the effects of open versus minimally invasive surgical technique in oncologic rectal resection with regard to postoperative muscle mass loss. In contrast to the upper gastrointestinal tract [12], the minimally invasive approach in the lower gastrointestinal tract is not a protective factor for the development of postoperative loss of muscle mass.

Because of the study design, no causal statements were possible. However, one conceivable cause is that open abdominothoracic oesophageal resection, unlike rectal resection, is a two‐cavity procedure that causes more severe perioperative trauma with prolonged immobilization. In addition, the formation of the gastric conduit massively alters the anatomy and absorption surface of the stomach [21], which potentially has a greater impact on eating behaviour and nutrient absorption postoperatively than rectal resection. Furthermore, oesophageal carcinoma is potentially more aggressive and, therefore, probably a more catabolic tumour disease than rectal carcinoma. This is supported by the poorer 5‐year survival data (15%–25%) for oesophageal cancer at all tumour stages compared with > 50% for rectal cancer [22]. However, the rate of preoperative sarcopenia in our unmatched cohort was even higher for rectal cancer (> 70%) than for oesophageal cancer (> 45%) [12].

Overall and DFS were not different between the MIS and open groups. The 3‐year OS was approximately 85% in both groups. Thus, our data confirm the results of the COLOR II study [23] on the oncological equivalence of both procedures. However, the 3‐year OS rate in the COLOR II study (70%–74%) was lower than in our study.

In this study, patients with a postoperative SMI loss > 10% had a significantly worse OS and DFS in our cohort. The 5‐year OS rate in patients without muscle mass loss was 93%, whereas it was only 44% in patients with muscle mass loss. Two meta‐analyses and recent studies have shown a significant correlation between sarcopenia and lower OS in patients with rectal cancer [10, 24]. There are indications that sarcopenia increases systemic inflammation and worsens survival rates [25, 26].

This difference in survival rates was associated with a significantly increased recurrence rate during the course of the disease in the sarcopenic group. Therefore, in this study, the development of postoperative muscle mass loss appears to be an expression of a more aggressive course of the tumour, with more frequent development of recurrence or metastases.

In the univariate analysis, several factors, such as age and ASA score, were found to be risk factors for postoperative muscle mass loss. However, in the multivariate analysis, only wound healing disorders were independent risk factors for the occurrence of postoperative muscle mass loss. From a biological perspective, cancer and its treatment lead to changes in metabolism, which cause both wound‐healing disorders and loss of muscle mass. Cancer‐related pro‐inflammatory cytokines and specific tumour factors, which trigger an energy‐intensive acute phase protein reaction, for example, the ubiquitin‐dependent proteasome pathway, drive the loss of skeletal muscle even in the presence of sufficient food intake. In addition, cancer leads to an increase in resting energy expenditure of up to 10% in around 25% of patients. A further 25% of cancer patients experience a reduction in their physical activity level for various reasons. This loss of physical activity leads to a deterioration and deconditioning of muscle mass. Together, these two factors lead to a vicious cycle with a progressive reduction in muscle mass and strength and ultimately to manifest sarcopenia. In addition, muscle plays an important role in protein metabolism and serves as a reservoir for amino acids, such as leucine or its metabolite, b‐hydroxy b‐methylbutyrate. These are necessary for both wound healing and muscle building [27, 28]. If metabolic shifts occur after cancer surgery, wound healing may initially be impaired, and sarcopenia may subsequently develop.

Screening for malnutrition and sarcopenia should be carried out at the time of diagnosis. Patients at risk identified in this way, as well as patients with an increased risk of developing wound healing disorders, such as smokers or diabetics, should then be integrated into special prehabilitation programmes prior to surgery. Such tri‐modal programmes consisting of sports training, nutritional therapy and psycho‐oncological support in the weeks before surgery can improve the postoperative outcome [29]. If a wound healing disorder occurs postoperatively, an extended screening for malnutrition should be carried out during the inpatient stay, and the daily caloric and protein requirements should be measured, for example, using bioelectrical impedance analysis or indirect calorimetry. On this basis, nutritional therapy should be initiated by specialized nutritionists and continued later in the outpatient setting at least until wound healing is completed.

Limitations of our studies are the relatively small number of cases including a monocentric Caucasian population. Therefore, our findings should be tested in randomized trials, and functional or biochemical markers of sarcopenia, such as bioelectrical impedance analysis and hand grip strength measurements, should also be used to substantiate the results.

## 5 Conclusion and Implications

In our study, we showed that, in contrast to oesophageal resection, oncological rectal resection does not result in pronounced postoperative skeletal muscle mass loss.

Compared with our initial hypothesis, MIS of the lower gastrointestinal tract is not a protective factor against the development of postoperative muscle mass loss compared with open surgery. However, two important findings were obtained: (1) Postoperative loss of muscle mass after rectal cancer surgery is significantly associated with worse OS, and (2) patients with wound healing disorders have a higher risk of developing postoperative loss of muscle mass. In summary, patients with delayed wound healing may require intensified nutritional support [30] to protect against sarcopenia and, thus, poorer long‐term oncological outcomes. This approach should be further evaluated through randomized interventional trials.

## Conflicts of Interest

The authors declare no conflicts of interest.

## Supporting information


**Table S1:** Patient characteristics, histopathologic and surgical findings.
