# Supplementary material for: Sarcopenia in Colorectal Cancer Surgery—Minimally Invasive vs. Open
Source: J Cachexia Sarcopenia Muscle. 2025 Sep 16;16(5):e70065. doi: 10.1002/jcsm.70065 (PMC12439183; doi:10.1002/jcsm.70065)
Supplement: Supplementary file 1 — Table S1: Patient characteristics, histopathologic and surgical findings. [file JCSM-16-e70065-s001.docx]

**Supplementary Table 1:** Patient characteristics, histopathologic and surgical findings

|  | **open** | **MIS** | **p-value** |
| --- | --- | --- | --- |
|  | **n = 74** | **n = 71** |  |
| **age [years]** | 61.3 (±11.4) | 61.9 (±10.8) | 0.837^#^ |
| **sex**  female  male | 20 (27.0)  54 (73.0) | 24 (33.8)  47 (66.2) | 0.470 |
| **BMI [kg/m^2^]** | 25.7 (±4.2) | 26.4 (±3.6) | 0.339^#^ |
| **ASA**  1  2  3  4 | 1 (1.4)  38 (51.4)  33 (44.6)  2 (2.7) | 8 (11.3)  43 (60.6)  20 (28.2)  0 (0.0) | 0.008 |
| **neoadjuvant treatment**  none  chemotherapy  radiotherapy chemoradiotherapy | 28 (37.8)  7 (9.5)  5 (6.8)  34 (45.9) | 47 (66.2)  2 (2.8)  0 (0.0)  22 (31.0) | 0.001 |
| **adjuvant treatment**  none  chemotherapy  chemoradiotherapy | 40 (54.1)  32 (45.9)  2 (2.7) | 38 (53.5)  33 (46.5)  1 (1.4) | 1.000 |
| **pT stage**  0  1  2  3  4 | 4 (5.4)  8 (10.8)  21 (28.4)  36 (48.6)  5 (6.8) | 4 (5.6)  8 (11.3)  27 (38.0)  31 (43.7)  1 (1.4) | 0.461 |
| **pN stage**  0  1  2 | 46 (62.2)  24 (32.4)  4 (5.4) | 39 (54.9)  22 (31.0)  10 (14.1) | 0.212 |
| **pM stage**  0  1 | 50 (67.6)  24 (32.4) | 65 (91.5)  6 (8.5) | 0.001 |
| **UICC stage**  1  2  3  4 | 20 (27.0)  15 (20.3)  15 (20.3)  24 (32.4) | 27 (38.0)  11 (15.5)  27 (38.0)  6 (8.5) | 0.002 |
| **loop ileostomy**  no  yes | 16 (21.6)  58 (78.4) | 15 (21.1)  56 (78.9) | 1.000 |
| **loop ileostomy closure**  <6 months  >6 months  never | 19 (25.7)  21 (28.4)  18 (24.3) | 24 (33.8)  22 (31.0)  10 (14.1) | 0.416 |
| **minor complications** | 11 (14.9) | 5 (7.0) | 0.186 |
| **major complications**  anastomotic leakage  bleeding  ureteral lesion  others | 18 (24.3)  16 (21.6)  1 (1.4)  0 (0.0)  1 (1.4) | 21 (29.6)  12 (16.9)  2 (2.8)  1 (1.4)  6 (8.5) | 0.575  0.532  0.615  0.490  0.059 |
| **CDC > 2** | 18 (24.3) | 18 (25.4) | 0.886 |
| **in hospital mortality** | 0 (0.0) | 0 (0.0) | 1.000 |
| **length of hospital stay [d]** | 16.6 (±11.9) | 15.2 (±11.0) | 0.059^#^ |

MIS, minimally invasive surgery; BMI, body mass index; ASA, American Society of Anesthesiologists Classification; CDC, Clavien-Dindo-Classification; n (%), mean (± standard deviation) or median (IQR), Fisher’s exact test, ^#^ Mann-Whitney U test
